# Pekin ducks are motivated to lay in their preferred nest substrate

**DOI:** 10.1017/awf.2023.19

**Published:** 2023-03-06

**Authors:** Lorelle Barrett, Shane K Maloney, Dominique Blache

**Affiliations:** 1 School of Agriculture & Environment, M079 and UWA Institute of Agriculture, The University of Western Australia, 35 Stirling Highway, Perth, WA 6009, Australia; 2 Animal Health & Welfare Directorate, Agriculture & Investment Services, Ministry for Primary Industries, PO Box 2526, Wellington 6140, New Zealand; 3 School of Human Sciences, M309, The University of Western Australia, 35 Stirling Highway, Perth, WA 6009, Australia

**Keywords:** animal welfare, behavioural demand, frustration, nest substrate, Pekin duck, preference test

## Abstract

Nest design is one factor contributing to floor-laying in farmed poultry. We investigated: (i) if ducks (*Anas platyrhynchos*) prefer a particular nest substrate; and (ii) how important that preference is to them, indicated by stress-induced hyperthermia, egg albumen corticosterone, and behaviour. Twelve female ducks that were trained in a push-door task had temperature data loggers implanted. Preference testing identified the most and least preferred nest substrates between sawdust, astroturf, and hemp fibres. A behavioural demand test then required the ducks to use push-doors to access nests containing either the most or least preferred substrate. The preferred substrate door was loaded with increasing weight (0–120% of bodyweight, four nights per workload) and eventually blocked to prevent nest access. The least preferred substrate door remained unweighted. The overall rank order of substrate preferences was sawdust > hemp > astroturf. Six of the 12 birds pushed all workloads and attempted to push the blocked door. The area under the curve (AUC) of hyperthermia was larger when the preferred substrate door was blocked compared with 0%. The AUC did not differ between nights 2–4 of the blocked door compared with night 1. Egg albumen corticosterone was unaffected. We conclude that laying Pekin ducks prefer manipulatable nest substrates and accessing one is important enough to pay a cost. The results indicate that a manipulatable substrate should be provided to commercially farmed nesting ducks.

## Introduction

Nest design is one of several factors contributing to floor-laying in farmed poultry, as nest features can determine if a laying bird finds the nest suitable (Appleby *et al*. 2004; Mench 2009). In chickens (*Gallus gallus domesticus*) and quail (*Coturnix japonica*), nest site selection can be influenced by the type of nest substrate available (Huber *et al*. 1985; Appleby & McRae 1986; Hughes 1993; Schmid & Wechsler 1998; Struelens *et al*. 2005; Guinebretière *et al*. 2012). In Pekin ducks (*Anas platyrhynchos*), the choice of nest can be influenced by enclosure and the presence of eggs (Makagon & Mench 2011; Makagon *et al*. 2011), but the importance of substrate to nest site selection has not been investigated.

Preference tests ask an animal to choose between different resource alternatives. Such tests have been used to establish preferences for nest substrate in a range of farmed animals. Chickens show preference for substrate that they can manipulate or peck at, such as peat or straw, rather than artificial substrates, such as plastic mesh or artificial turf (Huber *et al*. 1985; Struelens *et al*. 2005). Quail prefer nests containing hay over astroturf, while chaff tends to be more acceptable than hay (Schmid & Wechsler 1998). In fish, captive male Nile tilapia (*Oreochromis niloticus*) prefer sandy substrate over stones or no substrate for spawning (Mendonça *et al*. 2010). Rabbits (*Oryctolagus cuniculus*) prefer substrates such as straw or fine fibre over wood-shavings, the latter being commonly provided as nesting material on rabbit farms (Blumetto *et al*. 2010; Farkas *et al*. 2018). The conduct and interpretation of preference tests can be confounded by factors such as familiarity, learning ability, environmental cues, or other conditions that can influence an animal’s choice (Kirkden & Pajor 2006; Fraser & Nicol 2011). Notwithstanding these issues, preference tests can provide valuable insight into what an animal favours. Studies such as those noted above clearly demonstrate that an animal’s preference for substrate can be tested, and that the provision of resources in farming systems can be optimised if they are based on those preferences.

However, preference testing provides no insight into how important an animal considers a resource. Knowing how motivated an animal is to use a preferred resource provides insight into whether providing, or not providing, a preferred option will enhance or diminish animal welfare (Dawkins 1983). Behavioural demand tests measure how important an animal considers a resource by asking an animal to work increasingly hard to gain access to that resource (Mason *et al*. 1998). The combination of preference and motivational testing has been used to assess the motivation of hens for a preferred level of nest enclosure (Kruschwitz *et al*. 2008), a hen’s willingness to work for different foraging substrate (Gunnarsson *et al*. 2000), and the amount of work that pigs (*Sus scrofa*) are willing to perform for access to various rooting materials (Pedersen *et al*. 2005).

The provision of a suitable nest to farmed ducks should encourage positive welfare in laying birds, because nesting behaviour is highly internally regulated (Mench 2009). The decision to use a particular nest could be considered an active, goal-directed behaviour, and the fulfilment of such behaviour can contribute to a positive affective state. Failure to achieve the goal may result in negative states, such as frustration (Mellor 2015). To understand the response of an animal to its environment (and the subsequent interpretation of affect) multiple physiological and behavioural variables can be measured, preferably using a ‘hands-off’ approach to avoid inadvertent stress and the confounding of data (Cook *et al*. 2000). In birds, the measurement of corticosterone in the egg, instead of corticosterone in the plasma (the latter of which requires physical handling and blood sampling) has been used as a non-invasive method to assess birds’ response to acute stressors such as handling, ambient temperature increases, and movement of hens between cages (Downing & Bryden 2008). Correlation between the concentration of corticosterone in plasma and that in albumen has been demonstrated (Downing & Bryden 2008). The use of corticosterone in egg albumen as an indicator of stress has been trialled once in Pekin ducks that were denied access to a nest site (Barrett *et al*. 2021). That study found that any stress experienced was not reflected in the level of corticosterone in the albumen. As that was the first such study in Pekin ducks, further trials are warranted to better determine if the corticosterone concentration in egg albumen might be a useful physiological indicator of stress.

Another useful tool to assess the physiological effect of stressors on an animal is change in core body temperature to detect stress-induced hyperthermia (SIH). Animals exhibit SIH in response to many stressors, such as handling (Bittencourt *et al*. 2015), prolonged restraint (Gray *et al*. 2008), tests of fearfulness (Pedernera-Romano *et al*. 2010), and social stress tests (Kohlhause *et al*. 2011). Pekin ducks exhibit SIH when they are unable to access an established nest site (Barrett *et al*. 2021), a response considered to be related to frustration.

Frustration can occur if a strongly motivated behaviour is thwarted, resulting in a negative affective state for an animal. Changes in behavioural patterns can be used to gauge an animal’s subjective experience. In chickens, behaviours that have been investigated for their potential association with frustration include pacing (Wood-Gush 1972; Mills & Wood‐Gush 1985), preening (a displacement activity) (Duncan & Wood-Gush 1972; Mills & Wood‐Gush 1985; Meijsser & Hughes 1989), comfort behaviours such as head-shaking, tail-wagging or feather-raising/fluttering (Duncan & Wood-Gush 1972; Mills & Wood‐Gush 1985), feeding and drinking (also considered displacement behaviours) (Mills & Wood‐Gush 1985; Meijsser & Hughes 1989; Sherwin & Nicol 1993a), feather-pecking (Dixon *et al*. 2008), and redirected pecking behaviour (Kuhne *et al*. 2011). Wing-flapping has been associated with frustration in pigeons (*Columbia livia*) (Terrace 1972). As far as it can be determined, behavioural indicators of frustration have not been explored in the Pekin duck.

In the Australian duck industry, breeder ducks are typically provided with substrate such as sawdust or wood chips in the housing and in the nests. It has been estimated that floor eggs account for 20% of egg production in Australian duck farms (Luv-a-Duck, personal communication 2013). A better understanding of the factors that contribute to floor-laying in ducks could assist the industry to reduce the incidence of floor-laying and the associated costs. It is thus relevant to explore whether Pekin ducks have substrate preferences and how important that preference is. Thus, the use of preference and behavioural demand techniques together seems a logical approach to determine the significance of a preference for nest substrate in ducks.

With this study we aimed to:

- Determine if Pekin ducks exhibit nest substrate preferences;
- Assess ducks’ motivation to access their preferred substrate by asking them to perform an operant task (weighted push-door) to access their preferred substrate; and
- Assess the response to the stress of restricted access to the preferred substrate, by looking at changes in core body temperature, egg albumen corticosterone, and behaviour.
- It was hypothesised that:
- Ducks will show a preference for one type of nest substrate above others;
- Ducks will perform increasing amounts of work to access their preferred substrate; and
- Even if a less preferred substrate is available for free, ducks will show signs of stress, likely due to frustration, when they are unable to access their preferred substrate.

## Materials and methods

### Ethical approval

The procedures and experimental design were approved by the University of Western Australia’s Animal Ethics Committee (RA/1/300/1397).

### Study animals and housing

Fifteen female Pekin ducks arrived at the research facility when they were 22 weeks old. They were sourced from a local free-range producer. The ducks were housed in two outdoor grassed pens (approximate pen dimensions: 12 × 4 m; length × width), with seven birds in one group and eight in the other. The two pens were adjacent, enabling visual contact between both groups of birds. Nest-boxes with an aspen-chip substrate were placed at the back of each pen. The annual mean maximum and minimum temperatures for the area were 25.0 and 14.3°C, respectively. Artificial lighting was provided in the pens to give a lighting schedule of 16 h light (0400–0800h): 8 h dark (2000h–0400h) for the duration of the experiment. The ducks received a standard daily ration of chicken layer pellets, as well as being free to forage within the pens. In addition to drinking water, an open water source (135 × 60 × 15 cm; length × width × depth) was provided in each pen to allow bathing. Individual ducks commenced sporadic egg-laying at approximately 25 weeks old, with all ducks in regular lay by 30 weeks of age. The ducks were weighed weekly, with the weight range being 4.8–6.1 kg upon arrival and 5.2–6.1 kg at the completion of the experiment.

### Training and habituation in the behavioural demand apparatus

**Figure 1.**
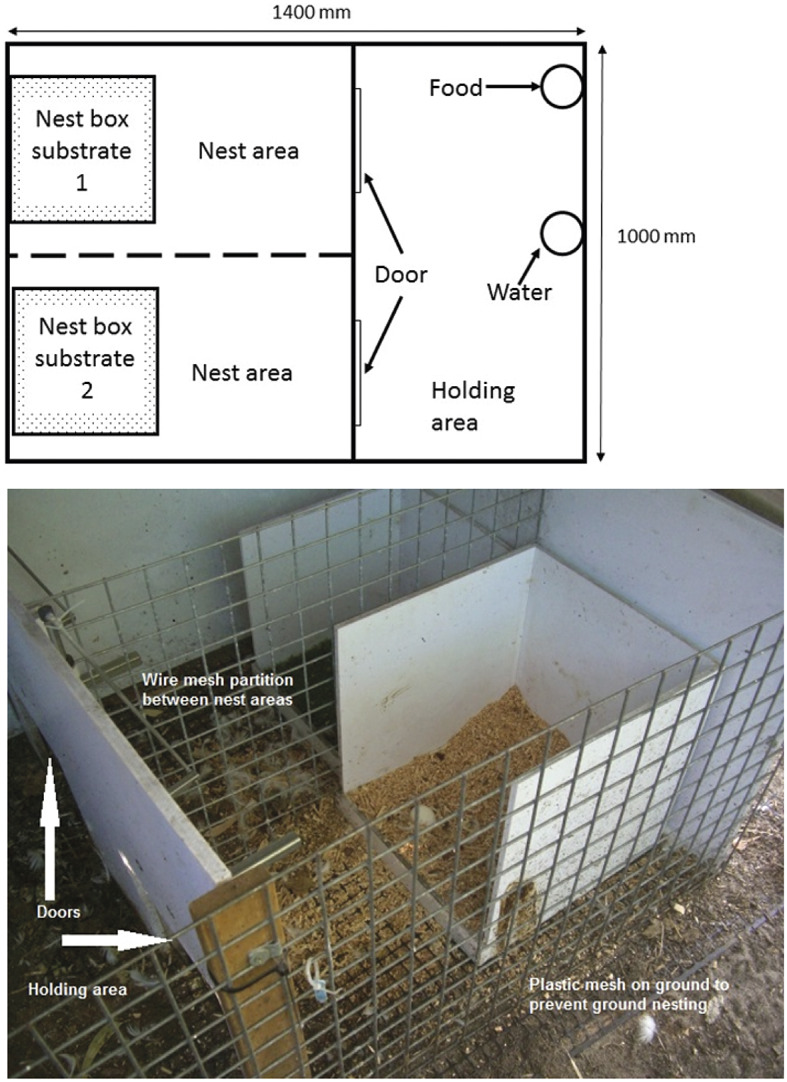
Showing diagram (top) and photograph (bottom) of the behavioural demand unit that was used to test the substrate preference of laying Pekin ducks, and their motivation to access a preferred substrate.

After arriving at the research facility, the birds had two weeks of habituation, as described in Barrett and Blache (2019). Handling was performed in a small temporary holding area for individual physical examination every second day during the first week, and then daily during the second week. Each pen had one empty behavioural demand unit (BDU) (Figure 1). During habituation, the end wall was removed from the unit, there were no nest-boxes in place, and the doors were open, so that the ducks could explore the unit unencumbered from the time of their arrival.

All 15 ducks underwent training to learn how to use the door of the BDU. For three consecutive days, on three times each day, each bird was held in one part of the unit and provided the opportunity to exit through the open BDU to return to her social group. On the subsequent three days, the process was repeated, but this time the birds were required to pass through the closed, unweighted door. Finally, for the last three days, the birds were required to pass through a weighted door (20% of the group average bodyweight) to return to their social group. Thus, each bird had 27 opportunities to learn to exit from the BDU. At all stages of the training, if the bird had not exited the BDU within 2 min, she was returned to the group until the next attempt. The 12 birds that had learned most effectively how to the use push-door were taken through the remainder of the experiment. The selection criterium for this decision was the 12 birds with the highest proportion of successful exits from the box when the door was closed (nine when the door was unweighted, and nine when the door was weighted with 20% weight, for a total = 18 opportunities to exit; see *Results; BDU training*). Social group was used as the training reward because it was easy to apply and has previously been demonstrated to be effective (Barrett & Blache 2019). No assumption was made about the relative intensity of motivation for the social group versus a preferred nest substrate.

Since the ducks were to be housed individually in a BDU overnight for preference and behavioural demand tests, they were progressively habituated to spend more time by themselves in a BDU. This habituation aimed to mitigate any stress due to social isolation that might occur and followed that outlined in Barrett *et al*. (2021). To briefly review, each bird was placed in the BDU for an increasing time over a period of six days, until they spent 3 h in the unit. Food and water were available in the unit during this time, and individuals were within sight and sound of their social group.

### Temperature data loggers

#### Surgical implantation

Surgery was performed when the ducks were 27 weeks old. Two days before surgery, the birds were moved indoors and housed in individual cages (870 × 740 × 600 mm [length × width × height], F-suite, Techniplast, Italy), to allow habituation to the indoor environment prior to surgery. A sterilised data logger (DST micro-T, Star ODDI, Iceland) was implanted into the body cavity using the surgical protocol described in Barrett *et al*. (2021). The following day, the birds were returned to a third outdoor pen for a two-week recovery period. The third pen was adjacent to the original two pens, and of the same construction and dimensions. Post-operative analgesia was provided for a further two days (0.5 mg kg^–1^ Metacam®, Boehringer, Australia). All birds recovered uneventfully.

#### Temperature data recording

Before implantation, the data loggers were programmed to record temperature every 5 min for the duration of behavioural demand testing. At the completion of the experiment, the ducks were humanely euthanased with pentobarbitone (150 mg kg^–1^ Lethabarb®, Virbac, Australia) and the data loggers were retrieved from the body cavity. A calibration test was then performed over 8 h at 33, 36, 39 and 42°C, before data were downloaded.

### Determining a preference for nest substrate

After a two-week surgical recovery period, four birds were placed in each of the three pens, with four BDUs in each pen. For one week, they were housed individually in a BDU overnight to habituate to a full night in the BDU. The BDU consisted of a holding area and two nest areas, each with an empty nest-box (Figure 1). Food and water were provided in the holding area. There was a wire partition between the nest areas so that the duck could still see out of the BDU, regardless of which nest area she was in, and the BDU was placed on a plastic mesh (Garden Master, Doncaster, Australia), that prevented the ducks from creating ground nests (Figure 1). After the week of habituation, preference testing commenced. Three nesting substrates were used to establish which was the most and least preferred by each individual bird. These were sawdust (WA & J King Pty, Martin, WA, Australia), hemp fibre (Mini-Hemp®, OzHemp, WA, Australia), and astroturf (Tuff Turf®, VIC, Australia). As we were keen to ascertain how the ducks ranked the three substrates against each other, the substrates were presented in pairs, rather than providing all three simultaneously. The three substrates provided six possible testing pairs, depending on whether a substrate was presented on the left or right side of the BDU (sawdust-astroturf, astroturf-sawdust, sawdust-hemp, hemp-sawdust, hemp-astroturf, astroturf-hemp; Table 1[a]). Substrates were presented on both the left and ride sides to account for lateralisation in any individual. Each pair was presented six times to each duck in a pre-determined order (Table 1[b]). This was achieved by placing a different pair in each of the four BDUs in a pen, with the ducks rotating through each of the four pairs over four consecutive nights. Four new pairs were then introduced. Eggs were collected every morning and the substrate that it was laid on (or floor if no substrate selection) was recorded. The choice of substrate was taken as the sole indicator of the duck’s nesting preference.

**Table 1(a).** Pairs for substrate preference test in laying Pekin ducks

Pair	Left	Right
1	A	H
2	H	A
3	S	H
4	H	S
5	A	S
6	S	A

‘Left’ and ‘Right’ refer to the side of the behavioural demand unit (BDU) that the substrate was presented on.

A = astroturf, H = hemp, S = sawdust.

**Table 1(b).** Order of presentation of nest substrate pairs. Four BDUs were placed in each of three pens, and each pen contained four ducks. The ducks rotated through the four pairs over four consecutive nights, then four new pairs were introduced into the pen

Nights	Pen 1		Pen 2		Pen 3	
1–4	1	2	5	6	5	6
	4	3	2	1	3	4
5–8	5	6	5	6	1	2
	2	1	3	4	4	3
9–12	5	6	1	2	5	6
	3	4	4	3	2	1
13–16	1	2	5	6	5	6
	4	3	2	1	3	4
17–20	5	6	5	6	1	2
	2	1	3	4	4	3
21–24	5	6	1	2	5	6
	3	4	4	3	2	1
25–28	1	2	5	6	5	6
	6	5	2	1	4	3
29–32	4	3	5	6	1	2
	2	1	3	4	4	3
33–36	5	6	1	2	5	6
	3	4	4	3	2	1

### Measuring the motivation of a duck to access a preferred nest substrate

Once the most and least preferred substrate had been identified overall, the behavioural demand testing began. For each test night, the BDU of an individual bird contained one nest-box with the most preferred and one with the least preferred substrate. To gain access to the nest with the most preferred substrate, the birds were required to perform increasing amounts of work as the door was incrementally weighted. The least preferred substrate remained ‘free’, with access via an unweighted door at all times. Each bird was placed into its BDU at approximately 1800h each evening, and the BDU was opened at approximately 0700h the following morning. Food and water were provided in the holding area. For the first four nights, the two doors were open, which allowed the birds to fully explore both nests. Workload then began at 0% of each bird’s bodyweight (BW) and increased in 20% increments every four nights until a maximum of 120% BW was reached. This artificial maximum cost was imposed because a previous study identified that higher workloads present a risk to the safety and welfare of the birds (Barrett *et al*. 2021). After the 120% workload had been completed, the preferred substrate door was blocked for four nights, so that the bird could still see the nest but was unable to pass the door to access it. A bird was considered to have failed a workload if the door was not passed for three of the four nights. A bird completed the experiment by either failing a workload or completing the final night of the experimental period. A video recorder above each pen recorded nightly activity in each BDU (Techview QV3034, Jaycar, Perth, WA, Australia). Behaviours were analysed from the footage using *Interact* behavioural analysis software (Interact, version 14.0, Mangold International, Arnstoff, Germany). The observation period began when the duck entered the BDU and ended 30 min after the duck passed the door (for workloads 0–120% BW) or after the last interaction with the door (for nights when the door was blocked). The location of each bird and any eggs laid was recorded each morning.

The behaviours that were analysed are outlined in Table 2. In addition to behaviours that were considered to indicate a duck’s motivation to access a nest site (looks/attempts to pass door, latency to pass preferred substrate door, latency to nest entry, and time spent in nest), actions that had previously been used to identify frustration in other bird species were included, as a first exploration of candidate behavioural indicators of frustration in Pekin ducks. The selected behaviours were pacing, preening, tail-shaking, feather-fluttering, head-shaking, wing-flapping, and pecking at the BDU structure. Feeding and drinking (considered displacement behaviours in other species) were not included, as the position of the feed/drink bowls relative to the camera meant that it was not possible to reliably quantify those behaviours from the video footage.

**Table 2.** Ethogram of behaviours of Pekin ducks during a behavioural demand test to assess their motivation to access a nest containing a preferred nesting substrate

Behavioural variable	Description
Looks through door	Duck passes bill or entire head through door (either preferred or least preferred substrate door) but does not push
Attempts to pass door	Duck places head and neck through door, shoulders are engaged with the door and duck is seen to exert effort and/or door is seen to move (either preferred or least preferred substrate door)
Latency to pass preferred substrate door	Time taken between duck’s first interaction with door to when it passes through the door
Latency to nest entry	Time taken between duck passing through preferred substrate door to first entering the nest
Time spent in nest	Total percentage of time spent in the preferred substrate during the 30 min after passing through the door
Tail shaking	Duck is seen to rapidly move tail up and down
Feather fluttering	Duck lifts and resettles feathers over the whole body, wings remain next to body wall
Wing flapping	Wings are extended out from body wall and rapidly moved up and down
BDU pecking	Duck is seen to peck or grab at parts of the BDU structure with its bill
Head shaking	Duck rapidly moves head from side to side
Pacing	Duck is repeatedly walking from one side of BDU to the other without pausing
Preening	Duck is engaged in grooming of feathers

The time at which a duck passed through a weighted door (at 0–120% workloads) was recorded from the video footage. The body temperature data within a time window of ± 15 min relative to the time the door was passed were analysed to detect SIH. For the blocked door, the association between SIH and the first attempt at the door was analysed for the first night. If a bird did not make any attempts at either door during the three subsequent nights of the blocked door, the association between SIH and the first look through the blocked door was analysed.

For each temperature data-point (T_c_), a smoothed value of temperature (T_s_) was calculated by averaging the temperature data for 12 h either side of each data-point. The standard deviation of T_s_ was also calculated across the same interval. Stress-induced hyperthermia was deemed to have occurred when the recorded T_c_ was 2.5× SD higher than T_s_ for that time-point. The duration of SIH was defined as the time interval over which consecutive data-points were 2.5× SD higher than T_s_ for that time. The area under the curve (AUC) for an SIH event was calculated by adding the temperature differentials (T_c_–T_s_) for the duration of an SIH event.

### Concentration of corticosterone in egg albumen

The concentration of corticosterone in egg albumen was measured using the radioimmunoassay described in Barrett *et al*. (2021). Briefly, eggs were collected daily and were considered to be associated with events two nights prior to the day of collection, based on the timeline of egg formation in laying birds (Johnson 2000). The albumen was separated from the yolk and frozen at –20°C until analysis. To extract corticosterone, the samples were thawed and a measured sub-sample was homogenised with distilled water by vortex. Diethyl ether was then added, the samples were vortexed for 10 min and then frozen at –20°C to allow separation of the aqueous phase. The solvent phase was then poured off and the diethyl ether was evaporated. Dried tubes were then covered and stored at 4°C until assay.

The corticosterone concentration in the extract was measured using the Immuchem double antibody corticosterone ^125^I RIA kit (MP Biomedicals, Orangeburg, NY, USA), using a modified procedure that improved sensitivity (Barrett *et al*. 2021). Radioactivity was counted using a Beckman Gamma counter. The assay has previously been validated using two quality controls; the coefficients of variation were 3.6% for 15.5 ng ml^–1^ and 6.5% for 90.1 ng ml^–1^, and the limit of detection was 5.2 ng ml^–1^.

### Statistical analysis

All 12 birds completed the preference test sequence however one bird was excluded from the results as she did not lay an egg for the first twenty nights of the preference testing, and did not lay in either substrate offered on the remaining nights. Six of the 12 birds completed all the behavioural demand workloads and attempted to pass the blocked door. Data from these birds are included in the analysis of behaviour and all analyses of egg albumen corticosterone. Owing to data loss from a hard drive, video footage of the blocked door nights from one bird could not be analysed. Consequently, only the five birds with complete data sets were used in the analysis of the AUC for hyperthermia. All data analyses were conducted in R statistical software (R Development Core Team 2017).

#### Determining a preference for nest substrate

For each of the eleven birds that were included in the data analysis, the substrate that an egg was in each morning was used to create a dominance matrix for the three substrates (Martin & Bateson 1986) (data not presented). The substrate on which the egg was found was considered the ‘winning’, or preferred, substrate of that pair, with the unchosen substrate being assigned as the ‘losing’ substrate. The rank order of substrate preferences for each individual bird, and for the overall outcomes, was determined using David’s Score, a ranking method that is used to determine dominance matrix hierarchies (Gammell *et al*. 2003).

#### Assessment of the importance of the preferred nest substrate

Descriptive statistics were used to investigate where ducks spent their time when they had free access to all areas of the BDU, and how many interactions (either an investigation or a visit) they had with nests that contained either the most or least preferred substrate.

To identify prospective relationships between behaviour and increasing workload or the door blockage, behavioural observations across nights of increasing door workload were explored graphically. Some of the measured variables were not normally distributed, and could not be normalised (looks through door, attempts at door, and all candidate behavioural indicators of frustration). Descriptive analysis was instead undertaken, as it was considered that the low number (n = 5) and power limited the use of non-parametric analysis. A baseline occurrence of each behaviour was calculated for each bird using the data from each of the four nights of 0% workload. These data were averaged, and the upper and lower limits of a 95% confidence interval were calculated. Behavioural indicators of frustration were first converted to incidence rates by dividing the frequency of the behaviour by the total time from the first door interaction to when the duck passed through the door to the preferred substrate for 20–120% BW, or until the last interaction with the door occurred on the nights when the door was blocked. The mean occurrence of a behaviour for each workload up to 120% BW (four-night average) and the blocked door (four-night average, and individual nights) was then compared against the bird’s baseline for that behaviour. Values that lay either above or below the 95% CI were identified, and a contingency table of the number of birds that lay below, within, or above their CI for each behaviour was created.

An ANOVA was performed on the latency to pass the door to the preferred substrate, the latency to nest entry, and the time spent in the nest. The data were log-transformed, where required, to normalise the distribution. Percentage data were converted to a proportion and arcsine-transformed prior to analysis. For each outcome variable, single explanatory variables (door workload, pen, and day) were tested for their association with that outcome. To account for repeated measures on individual birds, an error term was included in the final model. Interactions between variables were also assessed and carried forward to the final model if significant. Bonferroni *post hoc* testing was undertaken to compare pair-wise differences if there was a main effect of door workload on the outcome variable.

#### Stress-induced hyperthermia

An ANOVA was performed to analyse differences in the AUC of hyperthermia between workloads from 0–120% BW and the blocked door. A separate ANOVA was used to analyse the AUC of hyperthermia for the four nights when the door was blocked. The outcome variable (AUC) was regressed against the explanatory variables of workload, pen, and night for comparison of all workloads, and against night and pen for the nights when the door was blocked. Explanatory variables that were significant were included in the final model. To account for repeated measures on individual birds, an error term was included in the final model. Interactions between explanatory variables were also explored and included in the final model if significant. Data were log-transformed to normalise distribution prior to analysis.

#### The concentration of corticosterone in egg albumen

An ANOVA was performed to determine if door workload or blocking the door to the preferred nest substrate affected the concentration of corticosterone in the egg albumen. Corticosterone concentration was individually regressed against the explanatory variables of door workload, pen, and night, and explanatory variables were included in the final model if significant. To account for repeated measures on individual birds, an error term was included in the final model. Interactions between explanatory variables were also tested and included in the final model if significant.

## Results

### BDU training

All 15 of the birds exited through the open door of the BDU 100% of the time. When the door was down but unweighted, successful exits were made a mean (± SD) of 74 (± 27)% of the time. When the door was weighted with 20% of the group average bodyweight, successful exits were made 91 (± 26)% of the time. Overall, the mean percentage of successful exits for the 12 birds that were selected to continue in the experiment was 92 (± 8)%.

### Determining preference for nest substrate

The overall rank order of substrate preference was sawdust > hemp > astroturf (David’s score 1.43 vs 0.88 vs –2.32; Table 3). Individual preference for sawdust over astroturf was clear-cut in most of the birds: nine of eleven birds chose sawdust on 87.5–100%% of nights offered, while the remaining two birds chose sawdust over astroturf 67 and 57% of the time. Hemp vs astroturf was similar, with eight of eleven birds choosing hemp 100% of the time, and the remaining three birds choosing hemp 57–87.5% of the time. Preference for sawdust over hemp showed greater variation, with individuals choosing sawdust over hemp between 50–100% of the time. Based on these findings, sawdust was the most preferred substrate and astroturf the least.

**Table 3.** Test matrix for nest substrate preference for all individual tests combined

		‘Lost’			David’s Score
		Sawdust	Hemp	Astroturf	
‘Won’	Sawdust	–	57	64	1.43
	Hemp	43	–	73	0.88
	Astroturf	6	11	–	−2.32

Values indicate the total number of times that a substrate in a row was chosen (‘Won’) by laying Pekin ducks over the alternative substrate shown in a column (‘Lost’).

### Location of egg-laying

The location where eggs were found is given in Table 4. Ducks that completed 0–120% workloads (n = 6), laid 96% (range 89–100%) of their eggs in the nest containing the preferred substrate before they were denied access. Of the ducks that stopped pushing the door to the preferred substrate (n = 6), 68% (range 13–83%) of their eggs were laid in that nest before they ceased pushing.

**Table 4.** Site of egg-laying by individual Pekin ducks during a behavioural demand test when they had to perform increasing amounts of work to access a nest box that contained a preferred substrate (saw dust) while the least preferred option (astroturf) remained free

		Bird											
Door	Night	1	2	3	4	5	6	7	8	9	10	11	12
Open	1	1	1	3	1	1	5	1	1	1	5	1	1
	2	1	1	3	X	1	5	1	1	X	5	1	1
	3	1	1	3	1	1	1	5	1	1	1	1	X
	4	1	1	1	1	1	X	1	1	1	1	1	1
0%	1	4	1	4	1	1	1	1	X1	4	1	1	1
	2	4	1	4	1	1	1	1	X	1	1	1	X1
	3	4	1	4	1	1	2	1	X	1	1	1	1
	4	4	X1	4	1	X1	1	1	1	X1	1	X	1
20%	1	5	X	4	1	X1	1	1	1	5	1	1	1
	2	5	5	4	1	1	1	1	1	1	1	1	1
	3	5	1	4	1	1	1	1	X1	1	1	1	1
	4	5	1	4	1	1	1	1	1	1	1	1	1
40%	1	1*	1	4*	1	1	1	1	X1	1	1	1	1
	2	1	1	2	X1	X1	1	1	1	X1	X1	1	1
	3	1	1	2	1	X	1	1	X1	1	X1	1	X1
	4	1	1	1	1	5	1	1	1	1	1	1	X1
60%	1	1	1	2	1	5	1	1	1	1	1	1	1
	2	1	1	2	1	5	1	1	1	1	1	1	1
	3	1	1	2	1	X	1	1	1	1	1	1	X1
	4	1	1	1	1	5	1	1	X1	1	1	1	5
80%	1	1	1	1	1	5*	1	1	5	1	2	1	5
	2	1	X1	2	1	5	1	1	5	1	1	1	5
	3	1	1	1	1	5	1	1	5	1	2	1	5
	4	1	1	1	1	5	1	1	X	1	1	1	5
100%	1	1	1	2	X1	5	1	1	5*	X	X1	1	5*
	2	1	1	1	1	5	1	1	X	1	1	1	5
	3	1	1	1	1	5	1	X1	5	1	X1	1	5
	4	1	1	1	1	5	1	1	5	1	X1	1	X1
120%	1	1	1	1	1	5	1	1	X	1	X	1	1
	2	1	1	1	1	5	1	1	X	X1	X	X1	1
	3	1	1	1	1	5	1	1	X	X	5	1	1
	4	1	1	1	1	5	1	1	X	5	5	1	1
Blocked	1	1	X	1	3	X	5	5	5	5	X	5	1
	2	1	3	1	3	5	5	X	5	5	5	5	1
	3	1	X3	1	3	5	5	5	5	5	4	5	1
	4	1	X3	1	X3	X	5	4	1	5	X	5	1

Key: The sites where an egg was located are indicated by the following numbers: 1) Nest box containing preferred substrate; 2) Floor of preferred substrate nest area; 3) Nest box containing least preferred substrate; 4) Floor of least preferred substrate nest area; 5) Floor of holding area. X indicates no egg was laid; X followed by a number indicates that a bird was found in a nest box but had not laid an egg. Shaded boxes for ID numbers 1, 3, 5, 8, 10, and 12 represent the point at which a bird was discontinued from the experiment when it stopped pushing through the door to access the nest-box that contained the preferred substrate at the previous workload. * indicates the night on which both doors were re-opened for discontinued birds, allowing nest access again.

### Motivation for the preferred nest substrate

**Figure 2.**
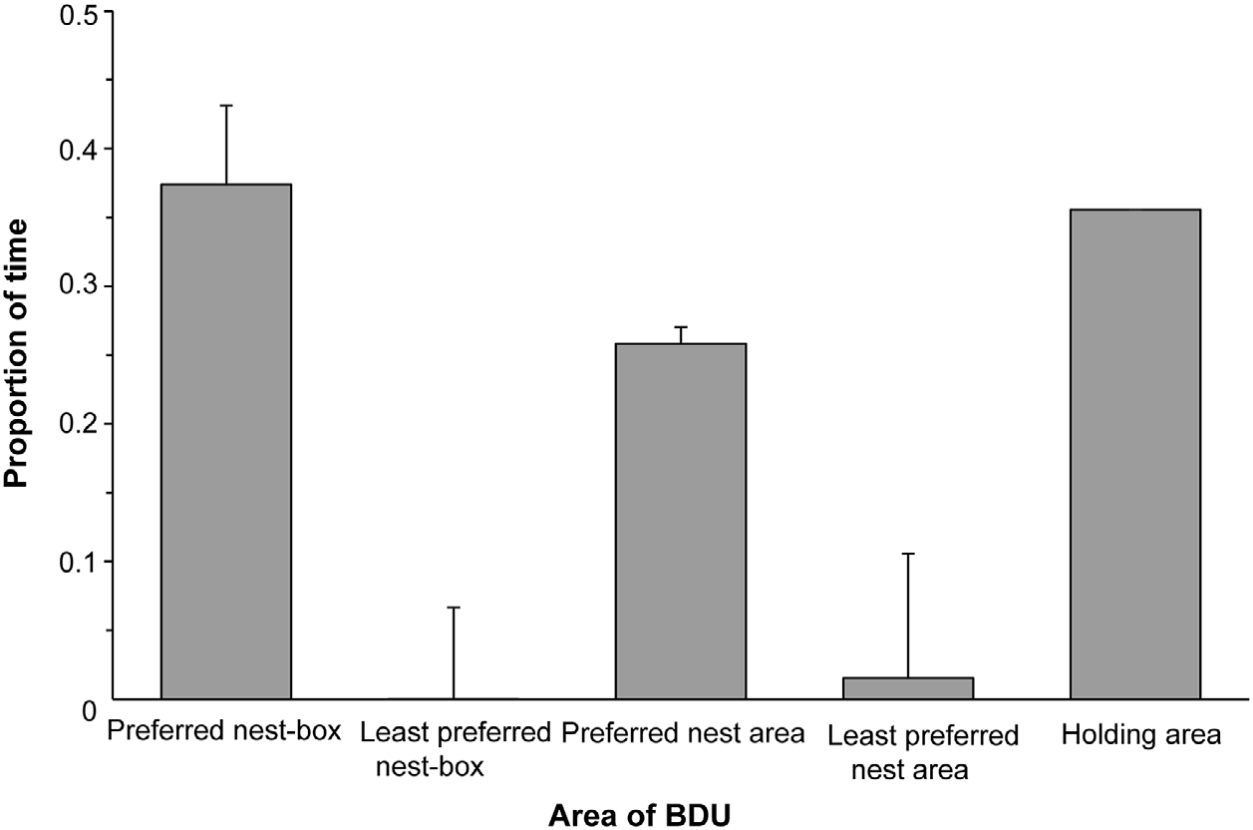
Proportion of time that the ducks (n = 11) spent in each area of the behavioural demand unit (BDU) on the four nights when both nests were freely available. Error bars show the standard error of the mean between birds.

**Figure 3.**
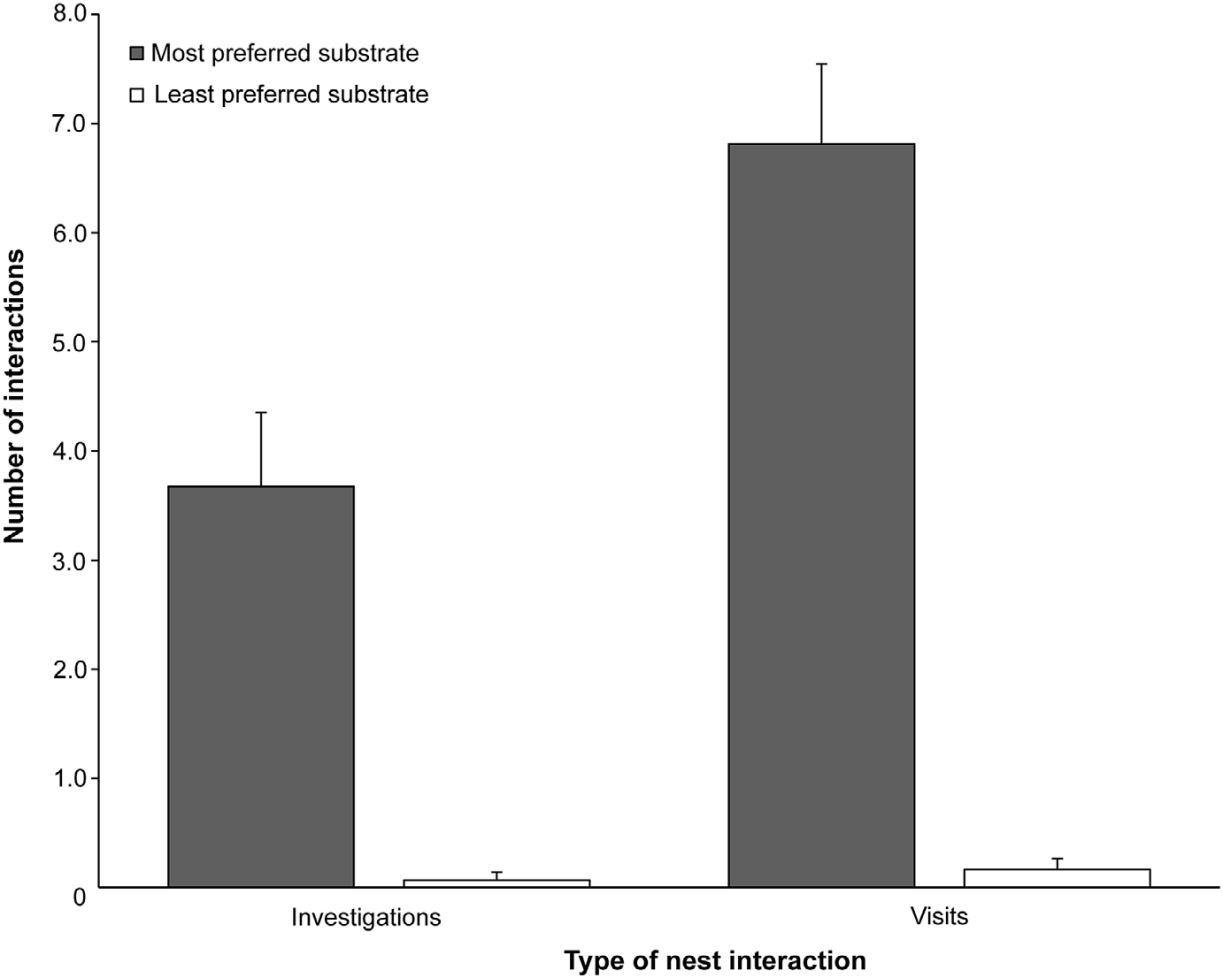
Number of interactions that ducks (n = 11) had with nests that contained either the most (sawdust; grey bar) or least preferred (astroturf; open bar) nesting substrate on the four nights when both nests were freely available. Error bars show the standard error of the mean between birds.

When the BDU doors were open, the ducks spent most of their time in the nest that contained the preferred substrate, or the surrounding area (37 and 26%, respectively; Figure 2). The ducks spent less than 5% of their time in or near the nest that contained the least preferred substrate (Figure 2). The ducks investigated the preferred nest more often (mean number over four nights: 3.67 vs 0.07) and had more visits to the nest that contained the preferred substrate than the one that contained the least preferred substrate (mean number over four nights: 6.1 vs 0.16; Figure 3).

As the door workload increased, there was no change in the proportion of time that was spent in the preferred nest-box, the latency to pass through the preferred nest door, or the latency to enter the preferred substrate nest. The overall mean (± SD) proportion of time spent in the preferred nest was 0.90 (± 0.23). The mean (± SEM) latency to pass the door to the preferred substrate was 115 (± 14) min. The mean latency to enter the preferred nest after passing through the door was 26 (± 3) s.

Six of the 12 birds completed all the workloads and attempted to pass the blocked door. Of the other six birds, two passed the door to the least preferred substrate at 0% on all four nights, one stopped pushing the door to the most preferred substrate after completing the 40% workload, two stopped after completing the 60% workload, and one stopped after completing the 100% workload. The four birds that stopped pushing the door to the most preferred substrate after completing 40–100% workloads then laid their eggs on the holding area floor and did not access the nest containing the least preferred substrate.

When the door was blocked, two of the six birds (birds 2 and 4) passed the ‘free’ door to the least preferred substrate within the first two nights (Table 4). A third (bird 7) passed the door to the least preferred substrate on night four of the blocked door. The remaining three birds (birds 6, 9 and 11) did not pass the door to the least preferred substrate, instead remaining in the holding area and laying their egg on the floor.

### Occurrence of hyperthermia

**Figure 4.**
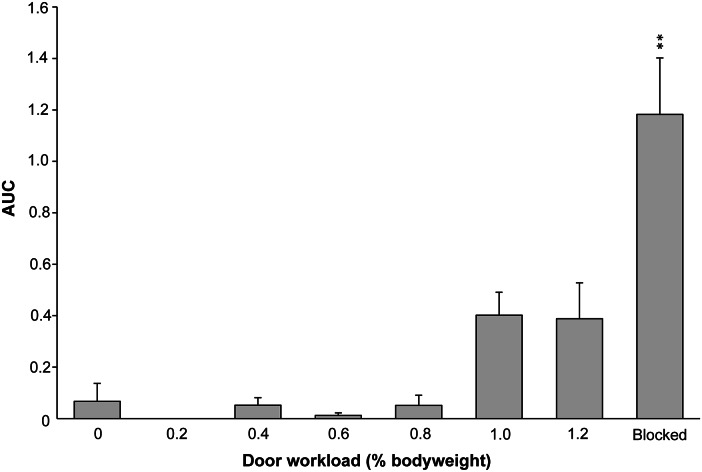
Area under the curve (AUC) of hyperthermia in Pekin ducks (n = 5) when they had to work harder to access their preferred nest site (0–120%) or when they were unable to access the nest (Blocked). Error bars show the standard error of the mean. ** Differ significantly from 0%; *P* < 0.001.

There was no difference in the AUC of body temperature at workloads up to 120%, when compared with 0%. There was also no difference between nights 2–4 of the blocked door when compared with the first night. The AUC of hyperthermia was larger when the door to the preferred substrate was blocked compared with 0% BW (mean AUC 1.18 vs 0.07°C × time [min], transformed model estimate = 1.36; *P* = 0.01; Figure 4).

Two of the six birds that did not complete all the workloads exhibited SIH on the nights when they first failed to pass the door. Bird 8 had an elevated T_c_ on all four nights of the 80% workload when she did not pass any door after interacting with the preferred substrate door. Bird 10 exhibited SIH on nights 1–3 of the 120% workload that she did not pass. The two birds that passed the door to the nest with the least preferred substrate did not exhibit SIH at all in response to their choice. Temperature data could not be collected or correlated with behaviour for the two remaining birds, as one’s data logger malfunctioned and ceased data collection part-way through the experiment, and an undetected camera fault resulted in behavioural footage not being recorded for the other.

### Behavioural observations

Of the six birds that completed all the workloads, four made more attempts to pass the preferred substrate door at 80% workload, and five made more attempts at 100 and 120% workload compared to their individual baseline values (Table 5). Up to the 120% level, all other behaviours were performed within the 95% CI of individual baselines by 50% or more of the birds.

**Table 5.** Number of attempts made by ducks (n = 6) to pass through a push door to gain access to a nest containing a preferred substrate

Bird	Baseline mean (95% CI)	20%	40%	60%	80%	100%	120%
2	1 (1, 1)	0	2.5	1	1.5	1	1.5
4	1 (1, 1)	1	1.5	2.3	2.3	4	4.3
6	1.3 (-1.9, 4.4)	1.8	2.8	2	2.8	4.5	4
7	1 (1, 1)	1	1.5	1.3	1	2.8	5.8
9	1 (1, 1)	1	1	1	2.7	1.3	12
11	1 (1, 1)	1.3	1	1	8.8	6.3	6.3

Shaded values indicate that the duck expressed the behaviour more often than their individual baseline value (mean + 95% confidence interval across all found nights of 0% workload).

When the door to the preferred substrate was blocked there were some changes in behaviour (Table 6). On the first night of the preferred substrate door being blocked, the number of looks through that door was higher than individual baselines for three of five birds (Table 6). The number of attempts at the preferred door was higher than individual baselines for all five birds on night 1 of the blocked door. During nights 2–4 of the preferred substrate door being blocked, the number of attempts at that door decreased for most birds, while the number of attempts at the least preferred door increased for three of five birds (Table 6). The rate of wing-flapping increased in two birds on night 1 of the blocked door, two birds on night two and one bird on night 3. The number of pecks at the BDU increased in three birds on night 1 of the blocked door, two birds on night 2, two birds on night 3 and one bird on night 4. Changes to rates of tail-shaking, feather-fluttering, head-shaking, pacing and preening were seen infrequently in some individuals on the nights when the door to the preferred substrate was blocked.

**Table 6.** Number of looks or attempts made by laying ducks (n = 5) when a push-door allowing access to a nest with preferred nest substrate was blocked, over four consecutive nights

			Night			
Behavioural variable	Bird	Baseline mean (95% CI)	1	2	3	4
Looks preferred substrate	2	10 (28, −8)	27	13	0	1
	4	5 (14, −5)	10	0	0	0
	6	7 (19, −5)	30	16	13	4
	7	5 (11, −2)	29	2	3	0
	11	10 (38, −18)	59	17	3	1
Looks least preferred substrate	2	6 (14, −3)	3	3	1	3
	4	2 (7, −3)	4	2	1	1
	6	1 (4, −2)	2	2	2	4
	7	2 (8, −3)	16	3	6	4
	11	3 (14, −8)	9	11	0	1
Attempts preferred substrate	2	1 (1, 1)	8	4	0	0
	4	1 (1, 1)	19	0	0	0
	6	2 (4, −2)	16	0	0	0
	7	1 (1, 1)	16	0	0	0
	11	1 (1, 1)	35	0	0	0
Attempts least preferred substrate	2	0 (0,0)	0	1	1	1
	4	0 (0,0)	1	1	1	1
	6	0 (0,0)	0	0	0	0
	7	1 (2, −1)	1	1	4	1
	11	0 (0,0)	0	0	0	0

Shaded values indicate that the duck expressed the behaviour more (darker shade) or less (lighter shade) often than their individual baseline value (mean + 95% confidence interval across all four nights of 0% workload).

### Concentration of corticosterone in egg albumen

The concentration of corticosterone in the egg albumen was not affected by the increasing workload, or the inability to access the preferred nest site (*F*_8, 290_ = 0.89; *P* =0.52). The mean (± SD) corticosterone concentration across all nights was 12.72 (± 4.47) ng ml^–1^.

## Discussion

The aims of the study were to determine if Pekin ducks display a preference for nest substrate, to assess the importance of that preference by asking them to exert increasing work to access that preferred substrate, and to assess their responses when they were unable to access their preferred substrate. The hypothesis that ducks have nest substrate preferences was supported by the results of the preference test, and the pattern of nest use and interaction when the birds had open access to a choice of substrates. The hypothesis that ducks are willing to work for their preference, and will show signs of stress when they are unable to access their preferred nest, was supported. The results of the behavioural demand test indicate that half of the ducks (6 of 12) were highly motivated to access the nest that contained their preferred substrate. The ducks that were willing to push 120% of bodyweight to access the nest experienced SIH and changed their behaviour when they were unable to access their preferred nest. However, any stress that occurred when they were unable to use their preferred nesting substrate was not reflected in changes to the concentration of corticosterone in egg albumen.

The overall rank order of substrate preferences was sawdust > hemp fibres > astroturf. Further evidence of substrate preference was found in the greater proportion of time that the birds spent in the nest-box that contained the preferred substrate (sawdust), or in the immediate area around this box, and the greater number of nest investigations and visits directed to this nest. One interpretation of these results is that, when offered a choice, Pekin ducks preferred to use a nest that contained substrate that could be manipulated. Similar preference for manipulatable nest substrate has been shown in chickens (Huber *et al*. 1985; Rietveld-Piepers 1987; Appleby *et al*. 1988). Farrowing sows also prefer nesting areas that contain straw over those that have structural elements of pre-formed nests (e.g. a hollow) but do not contain substrate (Arey *et al*. 1992).

In chickens, it is thought that fewer nest investigations of longer visit duration are indicative of preference, whereas higher numbers of nest investigations may indicate doubt (e.g. Meijsser & Hughes 1989; Struelens *et al*. 2008). As this is the first study of nest substrate preference in ducks, it is not yet clear if our findings are typical for this species. However, we note that once a duck passed through the closed door, she spent 90% of the observation period in the preferred nest regardless of door workload. This suggests that ducks were quite settled once in the nest-box. The low use of the astroturf nest when access to the sawdust nest was blocked would also support the notion that the low number of investigations and visits were indicative of the aversiveness of astroturf.

The results from the preference testing indicate that of the two manipulatable substrates, the ducks generally preferred sawdust over hemp fibres, though variations between individuals existed. The reasons for the variation are not entirely clear, but may include differences in particle size (hemp fibres being longer and larger), scent, or perhaps familiarity due to previous exposure to sawdust in the early stages of on-farm rearing. The issue of familiarity in preference testing is well recognised: animals may be attracted to, or avoid, environments that they have had previous experience with, though this may change over time as they become familiar with other available options (Fraser & Nicol 2011). It should also be noted that the presence of aspen chips in the nest-boxes, early on in the experimental period, could have contributed to the formation of a preference in the ducks, being somewhat similar to sawdust in particle size. Whilst it is difficult to know whether previous experience with sawdust or aspen chips influenced the initial choices made by ducks in the preference test, the repeated exposure to all substrates, and individual variation for choice of sawdust over hemp, suggest that familiarity was not the key driver in ducks choosing sawdust overall. A larger differential between sawdust and hemp, as well as sawdust and astroturf, would have been expected if previous experience with sawdust had an influence on a duck’s preference.

Most of the ducks were motivated to use a preferred nest substrate. Six of the 12 birds completed all workloads and attempted to pass the door to the preferred substrate even when it was blocked. Another four birds were willing to overcome workloads of between 40–100% bodyweight for access to the preferred substrate. For those ducks that completed all workloads, SIH was first recorded when they were required to push 100% of their bodyweight, a result similar to a previous study (Barrett *et al*. 2021), where 80% was the first workload at which SIH was observed. It is possible that the SIH seen at 100, 120% and the first night of the blocked door was due in part to the heat production that is associated with physical exertion. However, physical exertion cannot explain the results on nights 2–4 of the blocked door, because the birds still developed SIH despite making fewer attempts at the blocked door, and more at the unweighted least preferred substrate door, which would have required much less effort. Thus, the results indicate that if a bird is highly motivated to use a preferred nest substrate, denying the bird access to that resource elicits a stress response. This is supported by the lack of difference in the AUC between the first night of the blocked door and the following nights 2–4. The two birds that stopped pushing at 80 and 120% also showed SIH on nights when they made no attempts at the door. This finding suggests that they remained motivated for the nest but had surpassed their physical ability to access it, but consequently experienced stress, most likely due to frustration, when unable to access the preferred substrate. Although the small sample size in this study places limitations on how widely these results can be extrapolated to farmed duck populations, these findings, in conjunction with those of Barrett *et al*. (2021) provide preliminary evidence that further exploration of SIH could be a useful indicator of frustration in the Pekin duck.

The behavioural observations that we made here can provide a baseline of information from which behavioural indicators of frustration in Pekin ducks can be further explored. A majority of ducks showed an increase in the number of attempts and looks through the door to the preferred substrate, and an increase in the rate of wing-flapping and BDU pecking on the first night of the blocked door. Increased rates of wing-flapping and BDU pecking were seen in several individuals across all nights of the blocked door. While these results should be interpreted with caution, owing to small sample size and the limited ability for a robust statistical analysis, they do provide information on changes in behaviour that might be useful indicators of frustration in ducks. These behaviours have been shown to be indicators of frustration in other bird species. In pigeons, wing-flapping is increased during the early stages of a discrimination task in birds that have not yet learnt to correctly predict the presence or absence of a reward (Terrace 1972). Re-directed pecking in hens increases under operant learning conditions, when the hens are required to learn reversal of a task, or the task is not rewarded at all (Kuhne *et al*. 2011). It is also interesting to note that food-deprived hens show increased aggression towards a conspecific when they can see, but not access, food. This aggression takes the form of pecking and gripping the other bird with its beak (Duncan & Wood-Gush 1971). It is worth considering that the ducks’ pecking behaviour in the BDU may have been a form of redirected pecking. There is substantial evidence to indicate that not rewarding a previously rewarded task leads to a state of frustration, and subsequent aggression between birds (Papini *et al*. 2019). If such behaviour is considered in a commercial farming context, it can be theorised that where competition for resources exists (food, preferred nests, mates), or the environment does not allow the expression of behaviours that animals are highly motivated to perform, then aggressive interactions or abnormal behaviours, due to frustration, can be expected. Competition for nests has previously been proposed as a contributing factor to floor-laying in Pekin ducks, with 57% of nest exits being associated with an aggressive encounter (Barrett *et al*. 2019). Further studies are warranted to explore if redirected pecking occurs in ducks subjected to other frustrating situations, and whether this manifests differently in individual vs social contexts.

The limited change in pacing in potentially ‘frustrated’ ducks contrasts with previous findings in hens, where increased pacing was thought to be an indicator of frustrated nesting behaviour (Wood-Gush 1972; Sherwin & Nicol 1993b; Yue & Duncan 2003; Cronin *et al*. 2012; Tahamtani *et al*. 2018). A possible reason for the limited change in pacing seen in our ducks may be the different method of data collection. The present study recorded the rate at which pacing occurred, whereas those other studies counted the number of steps taken by birds. Thus, it would be useful in future to assess the step count of ducks in potentially frustrating situations to better align the interpretation of data with that in other species.

Displacement preening has been associated with frustration in hens (Duncan & Wood-Gush 1972). In our ducks there was no change in preening behaviour at any stage of the experiment. Preening may not be a useful indicator of frustration in ducks, or frustration may not have occurred. It is possible that due to the small sample size, and the generally low frequency at which preening occurred, an accurate representation of displacement preening was not gained in our experiment. The typical diurnal pattern of preening in Pekin ducks has yet to be established. Thus, preening as a possible indicator of frustration in ducks requires further exploration.

While we acknowledge the limitations of sample size, it is interesting to note the association between nest use and SIH when the ducks were exposed to the blocked door. Birds 2 and 4 chose to enter the least preferred substrate nest on either night 1 (bird 4) or 2 (bird 2) of the blocked door, and all subsequent nights thereafter. The SIH decreased in those two birds after night 2. Bird 7 exhibited SIH on all four nights of the blocked door, but chose to use the least preferred astroturf on the last night, while birds 6 and 11 exhibited SIH on all nights of the blocked door, and never used the alternative nest. There are two possible explanations for these differences. The fact that birds 6 and 11 chose never to use the least preferred substrate might be because their failure to pass the blocked door was such a negative experience that it impacted their willingness to try the alternative door. Similarly, in a previous study, ducks made no further attempts to pass a blocked door after failing at it on the first night (Barrett *et al*. 2021). An alternative explanation is that birds 2, 4, and 7 elected to use the least preferred substrate on the basis that it was the most adequate option available to them. These birds showed SIH on the first night that the door to sawdust was blocked and used the astroturf, suggesting that on that occasion they were frustrated by not being able to access the preferred substrate. However, the lack of SIH in birds 2 and 4 on subsequent nights suggests that, to them, there was no longer any frustration and that astroturf was better than laying on the floor. Thus, it may be that the astroturf was considered the ‘best of the worst’ nest substrate on offer. In contrast, birds 6 and 11 may have viewed the astroturf as equally, or more undesirable, than the floor, and never passed the alternative door. The diminished SIH in birds 2 and 4 could be explained by the process of habituation, where an animal’s response to a stressor decreases with repeated exposure. If habituation did occur, this suggests that birds 2 and 4 showed greater resilience about their choice of nest substrate than did birds 6 and 11. Possible explanations for an individual’s resilience may include genetic predisposition, broader early life experience, or variations in individual personality traits.

Although some ducks used the astroturf nest on occasion, the overall acceptability of this substrate was low. Besides being the lowest ranked substrate in the preference test, six of the 12 birds never used the astroturf nest during the behavioural demand tests. From a management perspective, astroturf is an attractive choice in poultry farming, because it is easier to provide and maintain than loose litter material. However, when making decisions about the type of resource to provide for animals, a straightforward consideration of whether animals will use that resource may be insufficient to meet welfare needs. Instead, the aim should be to provide resources that encourage a positive affective state by allowing the full expression of behaviours that an animal is motivated to perform. The expression of nesting behaviour is affected by the type of substrate that is provided to an animal. For example, chickens express nesting behaviour most fully when they are provided peat, compared to astroturf or wire mesh, with wire mesh resulting in a restless pattern of nesting behaviour (Struelens *et al*. 2008). In farrowing sows, the provision of straw allows for the most complex expression of nest-building and reduces stereotypic behaviour, compared to peat or no substrate at all (Rosvold *et al*. 2018).

In Australian duck production systems, it is typical for the barn floor and nest-boxes to be lined with some form of manipulatable substrate, with nesting bowls within the boxes (L Barrett, personal observation 2013). However, automated duck nests that are available internationally use either astroturf nest pads (Potters Poultry, Rugby, UK) or soft rubber mats (Vencomatic Group, Meerheide, The Netherlands). Given the strong preference for manipulatable substrates that was shown by ducks in the present study, the commercial availability of non-manipulatable substrates in large automated systems, and evidence in other species of the impact that substrate has on the expression of nesting behaviour, future studies should investigate whether the expression of duck nesting behaviour is influenced by the type of nest substrate that is available.

The analysis of corticosterone concentration in egg albumen was consistent with the results of Barrett *et al*. (2021), where no differences were found due to either increasing workload or psychogenic stress. Based on the findings of the two studies together, it is concluded that although changes in body temperature indicated that a stress response was more likely to occur with increased physical exercise and the inability to access a preferred nest site, the systemic corticosterone did not reflect that stress. It is possible that any change in corticosterone was not large enough and/or long enough to be reflected in the concentration of corticosterone in the albumen.

### Animal welfare implications

Access to a preferred nest substrate carries a high level of importance for ducks. Taken together with previous findings (Barrett *et al*. 2021), the results indicate that highly motivated birds that cannot use a suitable nest experience stress, most likely due to frustration. Given the strong internal motivation for nesting behaviour, the welfare of female breeder ducks in commercial farming systems could be negatively impacted if this behaviour is thwarted, due either to provision of unacceptable nest substrates, or competition for preferred substrates.

## Conclusion

Laying Pekin ducks exhibit a preference for manipulatable nest substrate. Astroturf was found to be generally undesirable to the birds for the purpose of nesting. Most ducks were willing to expend increasing effort to pass through a push-door to gain access to the preferred nesting substrate, and they exhibited SIH when they were unable to do so. Changes to behavioural indicators suggest that the ducks experienced some frustration and provide an initial reference point for future studies of frustration-related behaviours in this species. We acknowledge that the small sample size makes statistical analysis challenging for some parameters. Similar to previous findings, the measurement of egg albumen corticosterone was not a useful indicator of stress in this situation.

The results indicate that in a commercial farming context a manipulatable substrate should be provided to nesting ducks. Practical considerations, such as the ongoing availability, hygiene, and cost of substrate are all valid issues that need to be considered. As this was the first report of ducks showing nest substrate preferences, further work is required to validate these findings and explore how attractive ducks find different substrates in commercial settings, and whether their preferences can help to mitigate floor-laying in commercially farmed flocks.
